# Hemi-methylated CpG sites connect *Dnmt1*-knockdown-induced and *Tet1*-induced DNA demethylation during somatic cell reprogramming

**DOI:** 10.1038/s41421-018-0074-6

**Published:** 2019-02-12

**Authors:** Songwei He, Fuhui Wang, Yixin Zhang, Jinlong Chen, Lining Liang, Yuan Li, Mengdan Zhang, Xiao Yang, Hongshen Pang, Yingying Li, Xiaofen Huang, Dajiang Qin, Duanqing Pei, Hao Sun, Hui Zheng

**Affiliations:** 1 CAS Key Laboratory of Regenerative Biology, Hefei Institute of Stem Cell and Regenerative Medicine, Joint School of Life Sciences, Guangzhou Institutes of Biomedicine and Health, Chinese Academy of Sciences, Guangzhou Medical University, 510530 Guangzhou, China; 2 Guangzhou Regenerative Medicine and Health-Guangdong Laboratory (GRMH-GDL), 510530 Guangzhou, China; 3 Guangdong Provincial Key Laboratory of Stem Cell and Regenerative Medicine, 510530 Guangzhou, China; 4 University of Chinese Academy of Sciences, 100049 Beijing, China; 5 Institutes for Stem Cell and Regeneration, Chinese Academy of Sciences, 100101 Beijing, China; 6 Shenzhen University, Nanhai. Ave 3688, 518060 Shenzhen, China

**Keywords:** Reprogramming, DNA methylation

## Abstract

The relationship between active DNA demethylation induced by overexpressing *Tet1* and passive DNA demethylation induced by suppressing *Dnmt1* remains unclear. Here, we found that DNMT1 preferentially methylated, but TET1 preferentially demethylated, hemi-methylated CpG sites. These phenomena resulted in a significant overlap in the targets of these two types of DNA demethylation and the counteractions of *Dnmt1* and *Tet1* during somatic cell reprogramming. Since the hemi-methylated CpG sites generated during cell proliferation were enriched at core pluripotency loci, DNA demethylation induced by *Tet1* or sh-RNA against *Dnmt1* (*sh-Dnmt1*) was enriched in these loci, which, in combination with Yamanaka factors, led to the up-regulation of these genes and promoted somatic cell reprogramming. In addition, since *sh-Dnmt1* induces DNA demethylation by impairing the further methylation of hemi-methylated CpG sites generated during cell proliferation, while *Tet1* induced DNA demethylation by demethylating these hemi-methylated CpG sites, *Tet1*-induced DNA demethylation, compared with sh-Dnmt1-induced DNA demethylation, exhibited a higher ability to open the chromatin structure and up-regulate gene expression. Thus, *Tet1*-induced but not *sh-Dnmt1*-induced DNA demethylation led to the up-regulation of an additional set of genes that can promote the epithelial-mesenchymal transition and impair reprogramming. When vitamin C was used to further increase the demethylation ability of TET1 during reprogramming, *Tet1* induced a larger up-regulation of these genes and significantly impaired reprogramming. Therefore, the current studies provide additional information regarding DNA demethylation during somatic cell reprogramming.

## Introduction

Two types of DNA demethylation have been reported. Ten-eleven translocation methylcytosine dioxygenase 1 (TET1) mediates active DNA demethylation by converting 5-methylcytosine (5mC) to 5-hydroxymethylcytosine (5hmC), and further to 5-formylcytosine (5fC), or 5-carboxylcytosine (5caC)^1^. However, the newly synthesized and un-methylated DNA strand is methylated by DNA methyltransferase 1 (DNMT1) both during and after the S phase^2^. Inhibiting DNMT1 or inducing a rapid proliferation results in global DNA demethylation, which is considered passive DNA demethylation^3^.

The reprogramming of mouse embryonic fibroblasts (MEFs) to induced pluripotent stem cells (iPSCs) is frequently used as a model for studying DNA methylation. The fact that the DNA methylation level decreases during reprogramming reasonably suggests that sh-RNA against *Dnmt1* (*sh-Dnmt1*) and *Tet1* play beneficial roles. Furthermore, *Tet1* can replace *Oct4*, which is also named *Pou5f1*, to promote reprogramming^4^. In addition, the generation of iPSCs is facilitated by *sh-Dnmt1*^5^, which is further enhanced by *sh-p53*-induced proliferation acceleration^6^.

TET1 occupies gene loci associated with both the maintenance and establishment of pluripotency in a NANOG-dependent manner^7^. In addition, DNA demethylation induced by *sh-Dnmt1* is also enriched at core pluripotency loci, such as the *Oct4*, *Nanog*, and *Esrrb* loci^6^. Thus, the two types of DNA demethylation might share some targets and counteract each other during reprogramming.

Because 5hmC is an intermediate in 5mC demethylation to cytosine, 5hmC has also been considered an epigenetic marker distinct from 5mC and that is important for the maintenance and re-gain of pluripotency^8,9^. The suppression of *Dnmt1* with *sh-Dnmt1* induces DNA demethylation by preventing the methylation of hemi-methylated CpG sites that are generated during cell proliferation. There is no intermediate during the demethylation induced by *sh-Dnmt1*. Thus, the two types of DNA demethylation might function differentially during reprogramming.

Vitamin C (Vc), which promotes reprogramming, does not significantly affect the function of *sh-Dnmt1*^6,10^, and the combination of Vc and *Tet1* reverses reprogramming to a basal or even lower level^11^. This phenomenon has been explained by the increased activity of TET1 and the impairment of the mesenchymal-epithelial transition (MET), which is a necessary step during the early stage of reprogramming^12^. However, MEFs lacking all three *Tet* genes fail to initiate MET during reprogramming^13^, suggesting that the relationship between DNA demethylation and reprogramming is highly complex.

DNMT1 has been suggested to have a higher ability to methylate hemi-methylated CpG sites than to methylate un-methylated CpG sites^14,15^. If TET1 has different abilities in demethylating hemi-methylated and full-methylated CpG sites, the relationship between the two types of DNA demethylation should be further explored. In addition, although Vc-promoted and Tet-dependent demethylation have been extensively explored^16–18^, how and to what level Vc regulates TET1 activity are not fully understood.

Therefore, by using MEF reprogramming as an experimental model, the relationship between the two types of demethylation and the influences induced by Vc were investigated at both CpG and gene levels.

## Results

### Passive and active DNA demethylation have similar targets

**Fig. 1:**
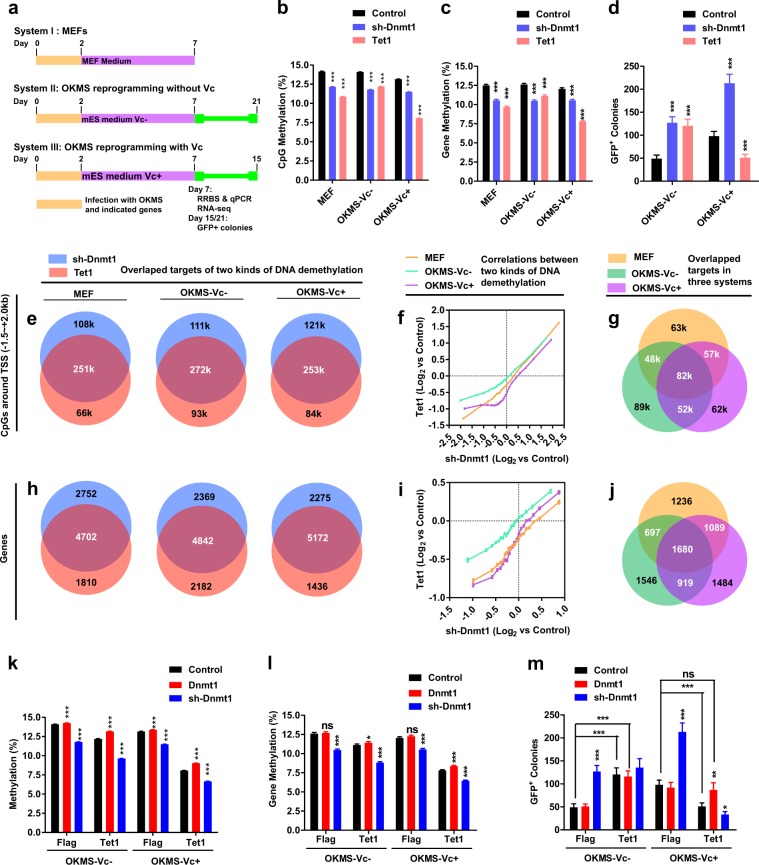
Two types of DNA demethylation share targets. **a** Schematic illustration of the three experimental systems used in the current studies. **b**–**d** The influences of *sh-Dnmt1* and *Tet1* on the methylation levels of CpG sites near TSS (−1.5 ~ + 2.0 kb) (**b**), methylation levels of all protein-coding genes (**c**), and iPSC generation (**d**) were summarized. **e–j** CpG sites (near TSS, −1.5 ~ + 2.0 kb) and genes with a larger demethylation than average were further selected. The overlapping targets of the two types of DNA demethylation were summarized in **e–h**. In addition, the correlations between demethylation induced by *sh-Dnmt*1 and *Tet1* were listed in **f** and **i**. Consistently demethylated CpG sites near TSS (approximately 82,000, **g**) and genes (1680, **j**) were summarized by overlapping the results shown in **e** and **h**. **k**–**m** *Dnmt1* and *sh-Dnmt1* were over-expressed with *Tet1*. The overall methylation levels of the CpG sites near TSS (−1.5 ~ + 2.0 kb) (**k**), overall methylation levels of all protein-coding genes (**l**), and iPSC generation (**m**) were summarized

MEFs were reprogrammed to iPSCs by exogenously expressing *Oct4*, *Klf4*, *c-Myc*, and *Sox2* (OKMS). In addition, to determine the relationship between passive and active DNA demethylation, *Tet1* and sh-RNA against *Dnmt1* (*sh-Dnmt1*) were used during reprogramming with (OKMS-Vc+) or without Vc (OKMS-Vc-) (Fig. 1a)^6,11^. Reduced representation bisulfite sequencing (RRBS) was used to monitor the changes on DNA methylation on Day 7.

RRBS assays were used to determine the methylation levels of more than 1.3 million CpG sites (approximately 6% of all CpG sites in the entire genome). Since the methylation levels of CpG sites near transcription start sites (TSS, −1.5 ~ + 2.0 kb) are more important for gene expression^6^, we focused on approximately 0.8 million CpG sites near TSS (approximately 45% of the CpG sites in these genomic regions). The methylation levels of approximately 14,500 protein-coding genes were determined by averaging the methylation levels of the CpG sites near corresponding TSS (−1.5 ~ + 2.0 kb).

As indicated in Fig. 1b–d, both *Tet1* and *sh-Dnmt1* induced significant DNA demethylation and promoted iPSC generation in the absence of Vc. Although *sh-Dnmt1* promoted reprogramming in the presence of Vc, *Tet1* inhibited iPSC generation under this circumstance. These observations are consistent with previous reports^4,6,11^.

Based on the modulations of reprogramming by *Tet1* and *sh-Dnmt1*, we anticipated a high-level overlap between the targets of these two types of demethylation during the reprogramming without Vc and little overlap in the presence of Vc. Surprisingly, the targets of these two types of DNA demethylation significantly overlapped in all three experimental systems, i.e., MEF, OKMS-Vc-, and OKMS-Vc + (Fig. 1e–j). Approximately 70% of the CpG sites that were demethylated by *Tet1* were also demethylated by *sh-Dnmt1* in all three experimental systems (Fig. 1e). The correlations between these two types of demethylation were also significant (Fig. 1f). In addition, approximately 82,000 CpG sites (approximately 10.1% of the 0.8 million CpG sites near TSS) were consistently demethylated by both *Tet1* and *sh-Dnmt1* in all three experimental systems (Fig. 1g).

According to the methylation levels of approximately 14,500 protein-coding genes, *Tet1*-induced and *sh-Dnmt1-*induced DNA demethylation in overlapping genes (Fig. 1h, i). In total, 1680 genes were consistently demethylated by both *Tet1* and *sh-Dnmt1* in all three experimental systems (Fig. 1j and Supplementary Table S1).

Since the targets of *sh-Dnmt1*-induced demethylation overlapped with those targeted by *Tet1*, the over-expression of *Dnmt1* might counteract *Tet1* and reverse its functions during reprogramming with Vc. Consistent with this hypothesis, we found that *Dnmt1* alone slightly increased DNA methylation and did not affect iPSC generation; however, *Dnmt1* impaired *Tet1*-induced demethylation and reversed its inhibitory effects on reprogramming in the presence of Vc (Fig. 1k-m). Furthermore, introducing *sh-Dnmt1* into MEFs boosted *Tet1*-induced demethylation and enhanced its inhibitory roles during reprogramming with Vc (Fig. 1k-m). Therefore, the two types of DNA demethylation have similar targets.

The additional 0.5 million CpG sites (outside −1.5 ~ + 2.0 kb of TSS) only covered a small portion of CpG sites in genomic regions, such as the gene body and intergenic region, suggesting that the overlap of the targets of these two types of demethylation could be observed in different regions. When all CpG sites (more than 1.3 million) detected in the current RRBS were analyzed, similar results were generated. Approximately 0.17 million CpG sites were consistently demethylated by both *Tet1* and *sh-Dnmt1* in all three experimental systems. The over-expression of *Dnmt1* counteracted with *Tet1* during reprogramming with Vc (Supplementary Fig. S1). Therefore, the two types of DNA demethylation share targeted CpG sites along the whole genome.

### Hemi-methylated CpG sites are preferentially demethylated by *Tet1* and *sh-Dnmt1*

When *Dnmt1* expression was suppressed by *sh-Dnmt1*, hemi-methylated CpG sites normally methylated by DNMT1 have higher chances of remaining intact and contributing to passive DNA demethylation. The hemi-methylated CpG sites generated during the S phase may be the shared targets of the two types of DNA demethylation. To test this hypothesis, the demethylation of hemi-methylated CpG sites was determined.

In the previous report^6^, we used whole genome bisulfite sequencing (WGBS) to analyze the methylation status of MEFs during G_1_ and G_2_/M phase (Supplementary Fig. S2a). The change in a particular CpG site becoming hemi-methylated and the enrichment of hemi-methylated CpG sites near TSS of a particular gene were considered enrichment of hemi-methylation and presented as absolute methylation differences (AMDs). The AMDs represent the absolute values of the methylation difference between positive and negative strands and were calculated as described in Materials and Methods.

**Fig. 2:**
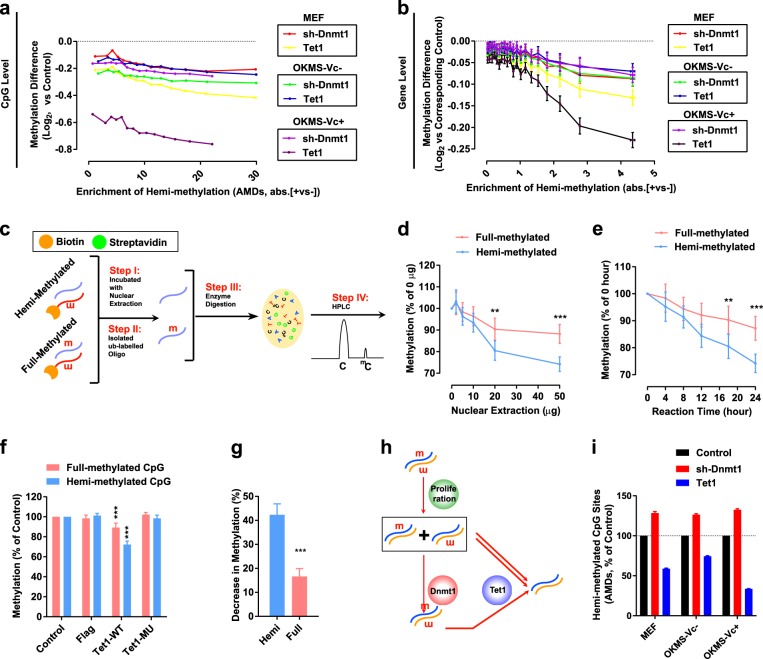
Hemi-methylated CpG sites are shared targets of the two types of demethylation. **a**, **b** CpG sites and genes were sorted according to the enrichment of hemi-methylation (AMDs between the positive and negative strands) and grouped into 14 and 20 groups, respectively. The demethylation of different groups was plotted against their enrichment of hemi-methylation (AMDs). **c** Schematic illustration of the in vitro model used to determine TET1 activity. **d**, **e** Dose-dependent (**d**) and time-dependent (**e**) curves of TET1-containing nuclear extraction to demethylate hemi- and full-methylated CpG sites. **f** Mutation of TET1 failed to induce demethylation in the current in vitro model. **g** The abilities of purified TET1 protein to demethylate hemi-methylated and full-methylated CpG sites. **h** Schematic illustration of the different demethylation induced by *sh-Dnmt1* and *Tet1*. **i** The abilities of *sh-Dnmt1* and *Tet1* to influence the amounts of hemi-methylated CpG sites

The calculated AMDs were significantly higher than the theoretical expectations at both CpG and gene levels (Supplementary Fig. S2b-c), indicating the existence of hemi-methylated CpG sites. Then, CpG sites near TSS (−1.5 ~ + 2.0 kb) and genes were sorted based on their AMDs (Fig. 2a, b). The demethylation induced by either *sh-Dnmt1* or *Tet1* increased along with the enrichment of hemi-methylation or AMDs (Fig. 2a, b), which is suggestive of the preferential demethylation of hemi-methylated CpG sites.

Since AMDs cannot distinguish un-methylated from full-methylated CpG sites, it was possible that CpG sites with low AMDs were originally un-methylated. But the methylation levels of the CpG sites or genes were relatively stable as the AMD increased, (Supplementary Fig. S2d-e). Therefore, this possibility was excluded.

Similar results were generated when the 20% CpG sites and genes with higher enrichment were compared with the remaining 80% of the CpG sites (Supplementary Fig. S2f-g). In addition, *Tet1* further demethylated the CpG sites and genes that had already been demethylated by *sh-Dnmt1* (Supplementary Fig. S2h-i). *sh-Dnmt1* boosted, but *Dnmt1* impaired, the abilities of *Tet1* to demethylate CpG sites and genes (Supplementary Fig. S2j-k). Such interactions between *Dnmt1* and *Tet1* were more significant in the 20% of CpG sites and genes with higher enrichment of hemi-methylation (Supplementary Fig. S2h-k).

Then, we determined the demethylation abilities of TET1 in an in vitro model. Single-strand oligonucleotides with CpG in the middle were labeled with biotin and used to form double-strand oligonucleotides with complementary and un-labeled oligonucleotides. The cytosines on the un-labeled oligonucleotides were always methylated, while the cytosines on the biotin-labeled oligonucleotides were methylated or unmethylated. Thus, we prepared double-strand oligonucleotides with hemi-methylated and full-methylated CpG sites. After incubation with TET1-containing nuclear extracts, the un-labeled oligonucleotides were isolated, purified, and digested into single nucleotides. The resulting 5 C and 5 mC were quantified by high-performance liquid chromatography (HPLC) (Fig. 2c). TET1 did have a higher ability to demethylate the hemi-methylated CpG sites than the full-methylated CpG sites (Fig. 2d, e). Mutating the catalytic sites of TET1 (H1652Y and D1654A) almost entirely blocked its demethylation activities (Fig. 2f).

Using purified TET1 protein to replace the TET1-containing nuclear extracts, similar results were observed. TET1 decreased the methylation of hemi-methylated CpG sites by more than 40%, while decreasing the methylation of full-methylated CpG sites by only approximately 16% (Fig. 2g). Therefore, TET1 has a higher ability to demethylate hemi-methylated CpG sites, and hemi-methylated CpG sites are shared targets of both types of DNA demethylation.

If *sh-Dnmt1* induces DNA demethylation by leaving more hemi-methylated CpG sites intact while *Tet1* induces DNA demethylation by converting hemi-methylated CpG sites to un-methylated CpG sites, the amount of hemi-methylated CpG sites should be differentially affected by *sh-Dnmt1* and *Tet1* (Fig. 2h). By comparing the enrichment of hemi-methylation (AMDs) in the current RRBS results, *sh-Dnmt1* increased, while *Tet1* decreased the amounts of hemi-methylated CpG sites (Fig. 2i). In addition, the decrease in the amounts of hemi-methylated CpG sites by *Tet1* in Fig. 2i was much larger than the percentage decrease of global DNA methylation in Fig. 1b, c, which further confirmed the preferential demethylation of hemi-methylated substrates by TET1.

### Expression changes are inconsistent with DNA demethylation

**Fig. 3:**
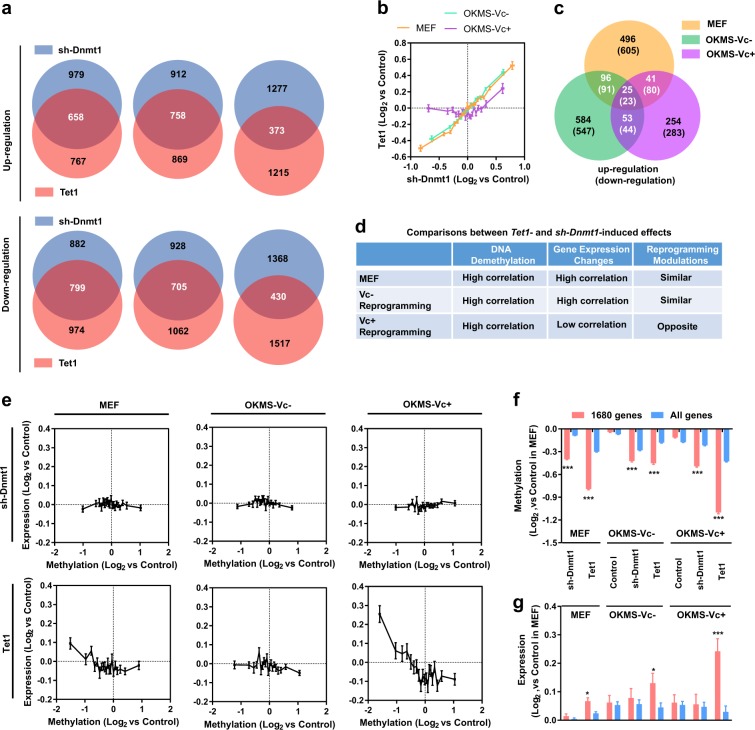
Expression changes induced by *sh-Dnmt1* and *Tet1* do not correlate well with demethylation. **a**–**c** Genes with more significant expression modulation than average (absolute values of expression changes of all genes) were selected. The overlapping genes between the *sh-Dnmt*1 and *Tet1* over-expressed groups were identified (**a**). In addition, the correlation between the expression changes induced by *sh-Dnmt*1 and *Tet1* were listed in **b**. Consistently up-regulated or down-regulated genes were summarized in **c** by overlapping the results shown in **a**. **d** *Tet1*-induced and *sh-Dnmt1*-induced effects on DNA demethylation, gene expression, and reprogramming were compared. **e** Correlations between demethylation and expression changes were determined in different groups. **f**, **g** The demethylation (**f**) and expression changes (**g**) between the consistently demethylated 1680 genes identified in Fig. 1g and all genes were compared

The expression changes induced by *Tet1* and *sh-Dnmt1* were compared in all three experimental systems. The expression changes induced by *sh-Dnmt1* correlated well with those induced by *Tet1* in MEFs and during reprogramming without Vc, while the correlation was impaired during reprogramming with Vc (Fig. 3a, b). In addition, the genes that were modulated consistently in all three experimental systems were rare, and only 25 up-regulated genes and 23 down-regulated genes were identified (Fig. 3c).

The three major downstream effects induced by *Tet1* and *sh-Dnmt1*, i.e., DNA demethylation, regulations of gene expression, and modulation of reprogramming, are summarized in Fig. 3d. *Tet1* and *sh-Dnmt1* induced overlapping DNA demethylation and similar expression changes and promoted reprogramming in the absence of Vc. In the presence of Vc, although *Tet1* and *sh-Dnmt1* also induced overlapping DNA demethylation, they differentially regulated gene expression and somatic cell reprogramming. Therefore, the different types of DNA demethylation may differentially affect gene expression.

To confirm the hypotheses mentioned above, the demethylation and expression changes were compared at the gene level in three experimental systems. The gene expression changes induced by *sh-Dnmt1* exhibited little correlation with DNA demethylation regardless of the experimental system analyzed (Fig. 3e). However, such correlation was significant in the *Tet1*-overexpressed groups, especially in the MEFs and during reprogramming with Vc (Fig. 3e). Thus, *Tet1*-induced demethylation has a higher ability to affect gene expression than *sh-Dnmt1*-induced demethylation. As indicated in Figs. 1j, 1680 genes were demethylated by *sh-Dnmt1* and *Tet1* in all three experimental systems. A comparison between these 1680 genes and all genes was performed to further confirm the above-mentioned hypotheses. Although both *sh-Dnmt1* and *Tet1* induced a larger demethylation in these 1680 genes, only *Tet1* induced a significantly larger up-regulation of expression (Fig. 3f, g).

### *Tet1*-induced active DNA demethylation has a higher ability to affect gene expression

One possible explanation for the different abilities of the two types of DNA demethylation to affect gene expression is that *sh-Dnmt1* induced demethylation by generating more hemi-methylated CpG sites, while *Tet1* induced demethylation by converting the hemi-methylated CpG sites to un-methylated CpG sites (Fig. 2h). We investigated how DNA methylation affects gene expression by analyzing chromatin accessibilities and gene expression in MEFs.

**Fig. 4:**
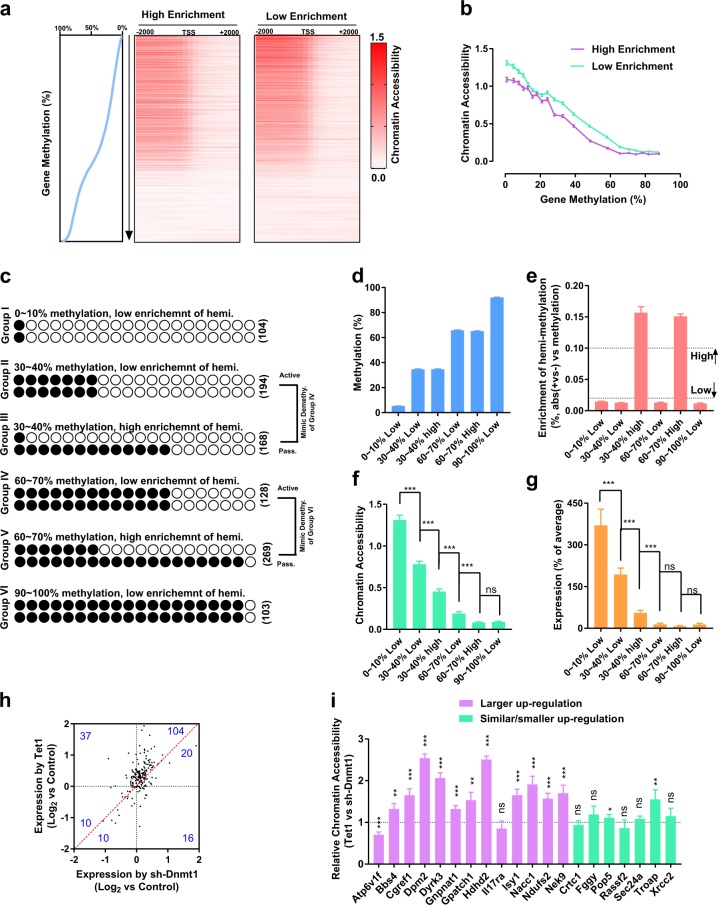
Two types of DNA demethylation differentially influence gene expression. The results obtained from the WGBS, ATAC-seq, and RNA-seq in assays of MEFs were analyzed together in **a**–**g**. The results obtained from WGBS (MEFs) and RRBS/RNA-seq (control, Tet1, and sh-Dnmt1 groups in MEFs) were analyzed in **h**, **i**. **a** First, genes were divided into 102 groups based their methylation levels (0%, 0–1%, …, 99–100%, and 100%). Then, the genes in each group were equally separated into two sub-groups, i.e., high enrichment and low enrichment, based on their AMDs. The genes from all high/low enrichment sub-groups were combined and sorted based on their methylation levels, and were presented along with their chromatin accessibility. The chromatin accessibilities generated from ATAC-seq were listed near TSS (−2.0 ~ + 2.0 kb). Gene methylation was plotted on the left. **b** First, the genes were divided into 20 groups (0–5%, …, and 95–100%). Then, the averages of gene methylation and chromatin accessibility were plotted. **c**–**g** Six sets of genes with particular methylation levels and enrichment of hemi-methylation were selected (**c**). The average methylation levels (**d**), enrichment of hemi-methylation (**e**), chromatin accessibilities (**f**), and gene expression (**g**) in MEFs were listed. The number of genes in each group was provided. **h**–**i** Of the 300 selected genes, the expression of 197 genes was detected in the current RNA-seq. The expression changes induced by *Tet1* and *sh-Dnmt1* were plotted in **h**. In total, 20 genes were randomly selected from these 197 genes, and the chromatin accessibilities of the genes in the *Tet1* and *sh-Dnmt1* groups were determined by ATAC-qPCR, compared, and plotted in **i**

WGBS and RNA-seq data of MEFs (GSE93417) were analyzed along with previously reported results (GSE93029) generated using an assay for transposase-accessible chromatin using sequencing (ATAC-seq) in MEFs. First, the genes were divided into 102 groups based on their methylation levels (0%, 0 ~ 1%, …, 99 ~ 100, and 100%). Then, the genes in each group were equally separated into two sub-groups, i.e., high enrichment and low enrichment, based on their AMDs. The genes from all high enrichment sub-groups were combined and sorted based on their methylation levels and were presented along with their chromatin accessibility in Fig. 4a. The genes from all low enrichment sub-groups were analyzed similarly. As shown in Fig. 4b, the genes were divided into 20 groups (0 ~ 5%, …, and 95 ~ 100%). The gene methylation and chromatin accessibility averages were plotted.

These results suggested that a negative correlation exists between chromatin accessibilities and DNA methylation at the gene level (Fig. 4a, b and Supplementary Table S2). In addition, when genes with similar methylation levels were separated according to their enrichment of hemi-methylation, those with higher enrichment had lower chromatin accessibilities (Fig. 4a, b). Therefore, genes with more hemi-methylated CpG sites have lower chromatin accessibilities, even if their methylation levels are similar.

Since the comparisons shown in Fig. 4a, b were performed with genes that have similar overall methylation levels, this analysis could not reflect the functions of *sh-Dnmt1*-induced demethylation. We selected several groups of genes (more than 100 genes per group) and compared their chromatin accessibilities and expression (Fig. 4c-e). Groups I, II, IV, and VI included genes with methylation levels of 0–10%, 30–40%, 60–70%, and 90–100%, respectively. Among the genes in these four groups, the ratios of AMDs to methylation levels were all below 0.02. Groups III and V included genes with methylation levels of 30–40% and 60–70%, respectively. Among the genes in these two groups, the ratios of AMDs to methylation levels were all above 0.1.

The genes in Group II and Group III were used to mimic the products of the genes in Group IV after active and passive DNA demethylation, respectively. Similarly, the genes in Group IV and Group V were used to mimic the products of the genes in Group VI after active and passive DNA demethylation, respectively. The comparisons suggested that both active and passive DNA demethylation resulted in chromatin opening and expression up-regulation, while active DNA demethylation resulted in a larger opening of chromatin and larger expression up-regulation (Fig. 4f, g). The different abilities of the two types of demethylation to successfully open chromatin might explain the different correlations between demethylation and the expression changes (Fig. 3e).

To confirm the above-mentioned hypothesis during actual DNA demethylation, the WGBS results of MEFs, RNA-seq and RRBS during DNA demethylation induced by *Tet1* and *sh-Dnmt1* were analyzed together. Then, 300 genes were selected by using the following criteria: (1) the methylation levels in MEFs were between 20–60%; (2) the methylation levels were decreased by more than 20% after the introduction of *Tet1* or *sh-Dnmt1*; and (3) the differences in the methylation levels between the *Tet1* and *sh-Dnmt1* groups were below 5% and no more than 10% of the average methylation levels in these two groups. Of these 300 genes, 197 genes were detected in the current RNA-seq. 124 genes underwent up-regulation after the introduction of *Tet1* or *sh-Dnmt1*, and 104 genes had larger up-regulation in the *Tet1* group than in the *sh-Dnmt1* group (Fig. 4h). In addition, of these 124 up-regulated genes, 20 genes were randomly selected. Of these 20 genes, 13 genes had larger expression up-regulation in the *Tet1* group, and 7 genes had similar or smaller expression up-regulation. Their chromatin accessibilities were determined by ATAC-qPCR instead of ATAC-seq. In total, 11 of the 13 genes have higher chromatin accessibilities in the *Tet1* group, whereas only 2 of the 7 genes have higher chromatin accessibilities in the *Tet1* group (Fig. 4i).

### Hemi-methylated CpG sites are enriched at core pluripotency loci

*Tet1* and *sh-Dnmt1* induced similar DNA demethylation and expression changes during reprogramming without Vc. However, since *sh-Dnmt1*-induced demethylation has a low ability to up-regulate genes and *Tet1* only induced low-to-medium levels of demethylation in this system, the current DNA demethylation did not correlate well with the up-regulation of gene expression. To understand how these two types of DNA demethylation promote reprogramming, the investigation should focus on the genes that exhibited higher demethylation after the introduction of *Tet1* and *sh-Dnmt1* into MEFs.

Since both *Tet1* and *sh-Dnmt1* preferentially demethylate hemi-methylated CpG sites, whether hemi-methylated CpG sites are enriched in certain loci was studied. The CpG sites near TSS (−1.5 + 2.0 kb) were grouped based on their methylation levels in MEFs and distance from surrounding CpG sites. As indicated in Supplementary Fig. S3a, the hemi-methylated status was enriched in the CpG sites whose distances from surrounding CpGs were between 45 and 90 bp. Therefore, hemi-methylated CpG sites might be enriched in genes whose TSS (−1.5 ~ + 2.0 kb) have approximately 40–80 CpG sites.

**Fig. 5:**
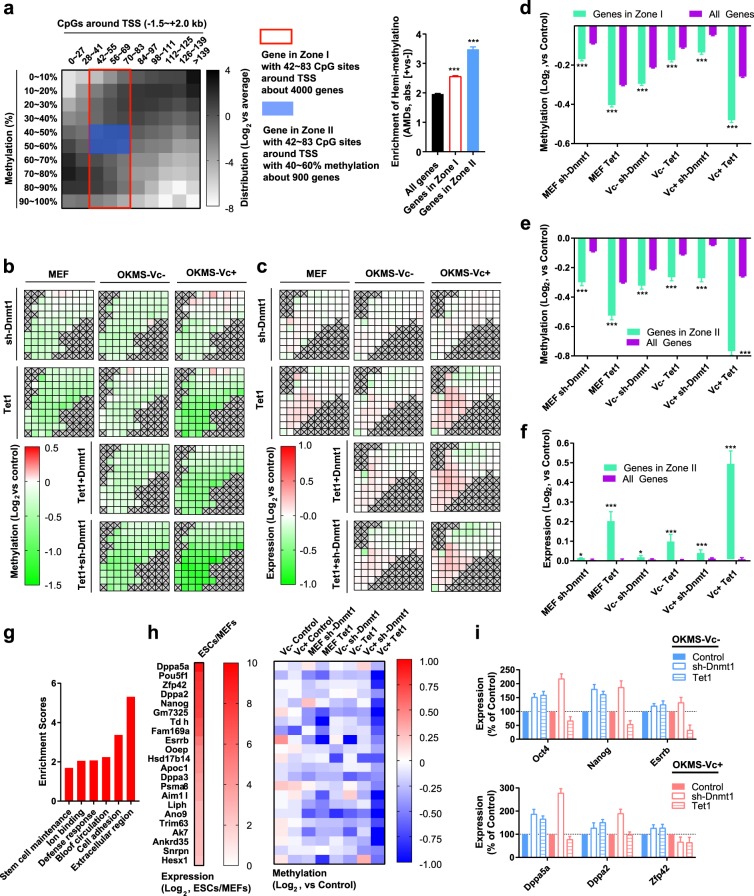
Demethylation of hemi-methylated CpG sites up-regulates pluripotent genes. **a**–**c** Approximately 20,000 protein-coding genes were divided into different groups based on their methylation levels and CpG densities near TSS (−1.5 ~ + 2.0 kb). The number of genes in the different groups were summarized in **a**. Genes with 42–83 CpG sites near their TSS (−1.5 ~ + 2.0 kb) were highlighted as in Zone I in **a**. Of the genes in Zone I, the genes with methylation levels between 40 and 60% were highlighted in Zone II. The changes in methylation of approximately 14,500 genes detected in the current RRBS were summarized in **b**, while the changes in the expression of approximately 11,000 genes detected in the current RNA-seq were summarized in **c**. Groups with less than 50 genes detected in RRBS or RNA-seq were not listed in **b** and **c**. **d**–**f** Demethylation of genes highlighted in Zone I and Zone II in **a** were compared to the average demethylation of all genes in **d**, **e**. The expression changes of 698 genes in Zone II were compared to the expression changes of all genes (**f**). **g**–**i** GO analysis results of the 698 genes in Zone II (**g**). The 22 genes that undergo significant up-regulation were selected and their demethylation was listed in **h**. Of these 22 genes, the expression changes of 6 pluripotent genes were determined by qPCR (**i**)

Then, we divided the protein-coding genes into different groups according to their methylation levels in MEFs and CpG densities near TSS (Fig. 5a). The changes in DNA methylation and gene expression after the treatment with *Tet1* and *sh-Dnmt1* were summarized in Fig. 5b, c, respectively. Genes with 42–83 CpG sites near their TSS (−1.5 ~ + 2.0 kb) were highlighted in Zone I (Fig. 5a).

Hemi-methylated CpG sites were also enriched in CpGs sites with methylation levels between 20 and 85% (Supplementary Fig. S3a). These CpG sites were observed in higher frequencies in genes with methylation levels between 20 and 60% (Supplementary Fig. S3b). In addition, based on the correlation between gene expression and methylation, similar levels of DNA demethylation induced larger expression changes in genes with methylation levels closer to 60% (Supplementary Fig. S3c). In summary, the two types of demethylation should preferentially up-regulate genes that have 42 ~ 83 CpG sites near their TSS (−1.5 ~ + 2.0 kb) and methylation levels between 40 and 60%. These genes were highlighted in Zone II in Fig. 5a. The genes in Zones I and II have more hemi-methylated CpG sites as demonstrated by the larger AMDs between the positive and negative strands (Fig. 5a).

The preferential demethylation and up-regulation were confirmed by analyzing the genes in Zones I and II (Fig. 5a). Approximately 5000 genes bear 42–83 CpG sites near their TSS (−1.5 ~ + 2.0 kb) and approximately 70% of these genes were detected in the current RRBS. These genes exhibited more DNA demethylation than other genes following the application of *sh-Dnmt1* o*r Tet1* (Fig. 5d). Of the approximately 800 genes in Zone II, 698 genes were detected in the current RRBS (Supplementary Table S3). These 698 genes exhibited significantly larger demethylation and up-regulation than the other genes (Fig. 5e, f).

The GO analysis suggested that among these 698 genes, the genes related to stem cell maintenance were enriched (Fig. 5g). Then, we identified 22 significantly up-regulated genes during reprogramming including *Dppa5a*, *Oct4*, *Zfp42*, *Dppa2*, *Nanog*, *Esrrb*, etc. (Fig. 5h). Their methylation levels were decreased by *sh-Dnmt1* and *Tet1* in all three experimental systems. *sh-Dnmt1-*induced and *Tet1*-induced demethylation alone could not up-regulate these genes in MEFs, but induced significant increases in these genes with the help of four Yamanaka factors in the absence of Vc (Fig. 5i). Therefore, *sh-Dnmt1-*induced and *Tet1*-induced demethylation is not strong enough to regulate gene expression, but might open the chromatin structure and allow access to Yamanaka factors.

Although not all methylation and expression changes in these 698 genes favored somatic cell reprogramming, the up-regulation of these genes which play critical roles in maintaining and regaining pluripotency, is enough to explain the promoted reprogramming by *sh-Dnmt1* and *Tet1* in the absence of Vc.

### Vc increases the abilities of *Tet1* to induce DNA demethylation

Subsequently, questions related to how Vc regulates these two types of DNA demethylation emerged. As indicated in Fig. 2a, b, the abilities of *Tet1* to induce DNA demethylation were higher during reprogramming with Vc than those during reprogramming without Vc, suggesting that Vc can potentiate DNA demethylation induced by *Tet1*. However, the *sh-Dnmt1*-induced DNA demethylation was unaffected (Fig. 2a, b). The abilities of Vc to potentiate *Tet1*-induced but not *sh-Dnmt1*-induced DNA demethylation were further confirmed by determining the global DNA methylation levels with HPLC, using purified TET1 protein in an in vitro activity assay, and calculating the demethylation activities based on the current RRBS and RNA-seq data (Supplementary Fig. S4).

**Fig. 6:**
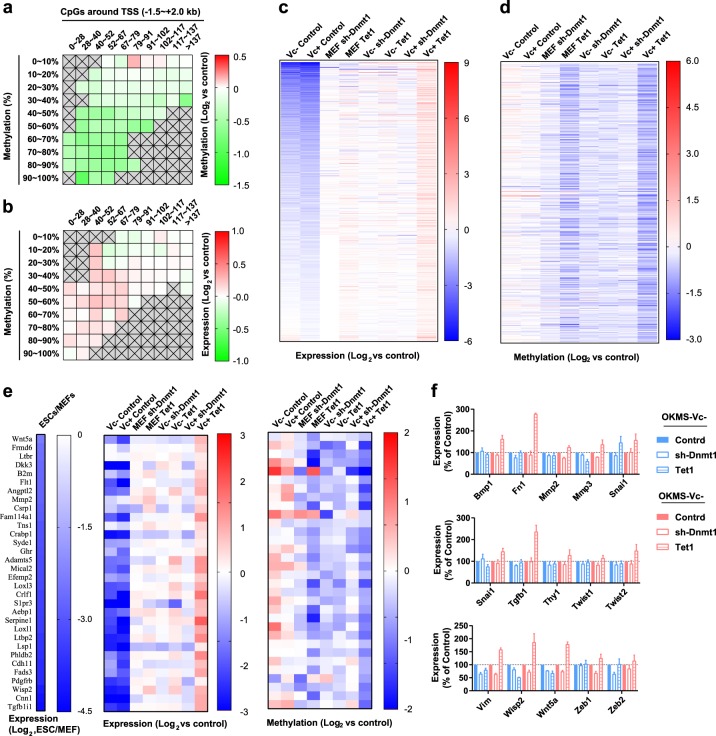
Demethylation of full-methylated CpG sites up-regulates mesenchymal genes. **a**, **b** The genes were divided as described in Fig. 5a. The differences in demethylation (**a**) and gene expression changes (**b**) induced by *Tet1* were compared between the two reprogramming systems. **c**, **d** In total, 606 genes were selected (see text). *Tet1* induced larger demethylation on these genes in the presence of Vc than in the absence of Vc. The expression of these genes decreased during reprogramming but could be reversed by *Tet1* in the presence of Vc. Demethylation (**c**) and gene expression changes (**d**) were summarized. **e**, **f** In total, 32 genes that undergo significant down-regulation were selected for analysis (**e**). Of these 32 genes, the expression changes of 15 mesenchymal genes were determined by qPCR (**f**)

Since Vc increased the abilities of TET1 to demethylate both hemi-methylated and full-methylated CpG sites, we compared the expression and methylation changes induced by *Tet1* in reprogramming with and without Vc. We observed increased demethylation and expression up-regulation (Fig. 6a, b).

Then, 606 genes were selected. These genes underwent more demethylation during reprogramming with Vc than during reprogramming without Vc and were up-regulated by *Tet1* during reprogramming with Vc (Supplementary Table S4). As indicated in Fig. 6c, d, *Tet1* demethylated and up-regulated these 606 genes in all three experimental systems.

We further selected 32 genes that were significantly down-regulated during reprogramming. Normally, the methylation of these 32 genes increased during reprogramming from MEFs to iPSCs. However, the over-expression of *Tet1* demethylated and impaired the down-regulation of these genes (Fig. 6e). When the demethylation activity of TET1 was further boosted by the presence of Vc, the increase in methylation levels and expression down-regulation of these genes were further reversed. The up-regulation of these genes was further confirmed by qPCR (Fig. 6f). Many of these 32 genes can induce the epithelial-mesenchymal transition (EMT), explaining the inhibitory roles of TET1.

Introducing *sh-Dnmt1* also induced demethylation in the 32 genes mentioned above, although *sh-Dnmt1-*induced demethylation was smaller than those induced by *Tet1* (Fig. 6e). The demethylation induced by *sh-Dnmt1* did not significantly affect gene expression (Fig. 6e, f), which was consistent with the fact that active DNA demethylation has higher abilities to affect gene expression than passive DNA demethylation.

TET1 demethylated the EMT genes less than the core pluripotent genes in MEFs and during reprogramming without Vc. In addition, without Tet1 or sh-Dnmt1, reprogramming process already resulted in demethylation of the pluripotent genes, whereas further methylation of the EMT genes (Figs. 5h, i and 6e, f). These effects of reprogramming process enlarged the difference in demethylation of the EMT and the pluripotent genes, and finally led to the different expression changes of these genes. However, during reprogramming with Vc, Vc treatment increased DNA demethylation of full-methylated CpG sites by approximately 2 folds, whereas increased DNA demethylation of hemi-methylated CpG sites by approximately 1.3 folds. Thus, the demethylation of the EMT genes increased more than the pluripotent genes (Figs. 5h, i and 6e, f), which led to the up-regulation of the EMT genes (Fig. 6e, f).

## Discussion

**Fig. 7:**
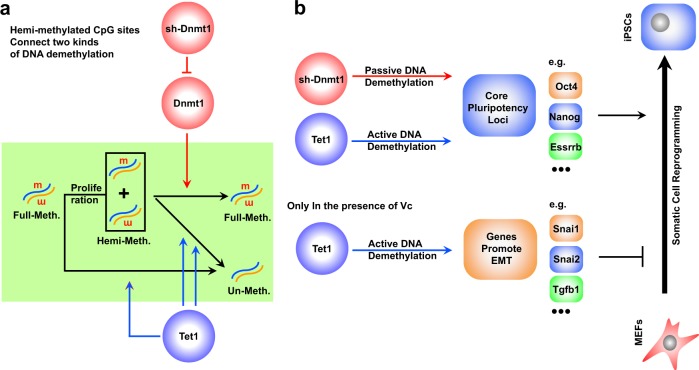
Passive and active DNA demethylation during reprogramming. **a** Suppressing *Dnmt1* with *sh-Dnmt1* induces passive DNA demethylation by impairing the methylation of CpG sites on strand newly synthesized during the S phase. The over-expression of *Tet1* induces active DNA demethylation by demethylating both hemi-methylated and full-methylated CpG sites. TET1 has higher abilities to demethylate hemi-methylated CpG sites than full-methylated CpG sites. **b** Both passive and active DNA demethylation were enriched at core pluripotency loci and promoted somatic cell reprogramming in the absence of Vc. Because of the higher abilities of active DNA demethylation than passive DNA demethylation to open the chromatin structure and regulate gene expression and the abilities of Vc to increase the demethylation activity of TET1, the over-expression of *Tet1*-induced demethylation genes that promoted the EMT, up-regulated these genes, and impaired reprogramming in the presence of Vc

Understanding the relationship between DNA demethylation and gene expression could facilitate the investigation of a variety of biological processes. In the current studies, the contributions of two different types of DNA demethylation to expression modulations and somatic cell reprogramming were determined (Fig. 7).

During reprogramming without Vc, s*h-Dnmt1*, and *Tet1* only induced low-to-medium DNA demethylation, because of the low ability of *sh-Dnmt1*-induced demethylation to induce gene up-regulation and relative lower expression up-regulation of *Tet1* by *Tet1*-encoding retrovirus, respectively. Since both *Tet1* and s*h-Dnmt1* exhibited a preference towards demethylating hemi-methylated CpG sites, demethylation was only significant in genes with high enrichment of hemi-methylation, such as, *Dppa5a*, *Oct4*, *Zfp42*, *Dppa2*, *Nanog*, and *Esrrb*, which are core transcription factors in maintaining pluripotency. In addition, although the demethylation induced by *Tet1* and s*h-Dnmt1* in these genes was insufficient to up-regulate them, the slight opening of chromatin allowed for exogenously expressed Yamanaka factors to function at these core pluripotency loci and subsequently promote reprogramming. Thus, *Tet1* and s*h-Dnmt1* induced similar modulations on gene expression during reprogramming with Vc.

In the presence of Vc, the abilities of *Tet1* but not s*h-Dnmt1* to induce DNA demethylation increased. In addition, the Vc treatment led to a 2-fold increase in the DNA demethylation activity of TET1 in full-methylated CpG sites, whereas a 30% increase was observed at hemi-methylated CpG sites (Supplementary Fig. S4b). Thus, demethylation induced by *Tet1* was not only significant in genes with a high enrichment of hemi-methylation, but also significant in genes related to the EMT (Fig. 6e, f). In addition, the larger demethylation induced by *Tet1* was sufficient to up-regulate gene expression. Therefore, in the current studies, genes related to the EMT were up-regulated and subsequently impaired reprogramming. In summary, when DNA demethylation induced by *Tet1* is weak and close to that during reprogramming without Vc, reprogramming towards pluripotency is promoted. However, when DNA demethylation induced by *Tet1* is strong, the EMT is promoted and reprogramming is impaired. Since the expression of markers of pluripotency and the EMT are highly associated with cancer, *Tet1* likely performs complex functions during cancer development and progression^19^. Furthermore, Vc has been suggested to kill KRAS and BRAF mutant colorectal cancer cells by targeting GAPDH^20^. Thus, it is also possible that Vc kills cancer cells by increasing the ability of TET1 to induce DNA demethylation.

We described the demethylation induced by *Tet1* as a conversion from full-methylated or hemi-methylated CpG sites to un-methylated CpG sites. Actually, such a process included multiple steps and intermediates that at a minimum include the conversion from 5mC to 5hmC and from 5hmC to 5fC or 5caC. The contributions of these intermediates to chromatin accessibility and gene expression are unknown. Therefore, additional investigations should be performed. In addition, in the current studies, 5mC was quantified in WGBS, RRBS, and HPLC assays. WGBS and RRBS assays use bisulfite to convert cytosine residues to uracil. Bisulfite sequencing does not distinguish 5mC from 5hmC and treats 5fC and 5caC as un-methylated cytosine^21,22^. The amount of 5hmC is approximately 10% of 5mC in the central nervous system and less than 4% in other tissues^23^. In addition, the amount of 5fmC and 5caC are both below 2% of the amount of 5hmC^23^. The difference between hemi-methylated and full-methylated CpG sites in the current paradigm should be the differences between H/C + M/C and H/H + H/M + M/M (H, M, and C refer to 5hmC, 5mC, and 5C, respectively). Our conclusions are based on the DNA demethylation of H/H + H/M + M/M to H/C + M/C. Although 5hmC might be a distinct state from 5mC, our main conclusion should not be affected. However, using HPLC to determine DNA methylation will digest DNA into single nucleotide. 5C and 5mC were recognized as 2′-deoxycytidine and 5-methyl 2′-deoxycytidine, respectively, while 5hmC, 5fC, and 5caC were not assayed. Therefore, the existence of 5hmC, 5fC, and 5caC does not significantly affect the current results.

## Materials and methods

### Primary culture of MEFs

MEFs were derived from E13.5 mouse embryos carrying the Oct4-GFP transgenic allele^24^. Briefly, the head, all internal organs, and the vertebral column containing the spinal cord were removed from the embryos. After washed with PBS twice, the embryos were dissociated with trypsin/EDTA and pipetting. The dissociated cells were grown to confluence in high glucose DMEM (Thermo Fisher), supplemented with 10% FBS (Excell), nonessential amino acids (NEAA, Thermo Fisher), and GlutaMAX (Thermo Fisher), diluted 1:10, and grown to confluence again. Then, MEFs were frozen at 5 × 10^6^ cells/ml for future experiments. The MEFs were confirmed to be mycoplasma-free before storage.

All procedures related to animal were performed in accordance with the National Institutes of Health Guide for the Care and Use of Laboratory Animals (NIH Publication No. 80–23) and approved by the Institutional Review Board of the Guangzhou Institutes of Biomedicine and Health, Chinese Academy of Sciences. All efforts were made to minimize the number of animals used and their suffering.

### Generation of iPSCs

MEFs were maintained in high-glucose DMEM (Thermo Fisher) supplemented with 10% FBS (Thermo Fisher), NEAA (Thermo Fisher), and GlutaMAX (Thermo Fisher). The retrovirus was produced using Plat-E cells, pMXs-based retroviral vectors, and a calcium phosphate transfection protocol. Within two passages, the MEFs were split into twelve-well plates (1.5 × 10^4^ cells/well). After the addition of polybrene to 4 μg/ml, the viral supernatant was used to infect the cells. Viruses encoding *Oct4*, *Klf4*, *c-Myc*, and *Sox2* were introduced into the cells twice, on Day 0 and on Day 1, and mES or mES-Vc (high glucose DMEM, NEAA, GlutaMAX, Leukemia Inhibitory Factor (PeproTech), 2-mercaptoethanol (Thermo Fisher), and 10% FBS without or with Vc (Sigma) was used on Day 2. The medium was replaced daily with freshly prepared medium.

Antibody against NANOG (R&D Systems, AF2729), REX1 (Abcam, ab50828), or FLAG (Sigma, F1804) was used. Proper secondary antibodies (Thermo Fisher, A110757 and A31573) were used.

### Quantitative RT-PCR (qPCR)

For the qPCR, total RNA was extracted from the cells using TRIzol (Thermo Fisher), and 5 μg of RNA were used to synthesize cDNA with ReverTra Ace® (Toyobo) and oligo-dT (Takara) according to the manufacturers’ instructions. The transcript levels of the genes were determined using SYBR Premix Ex Taq II (Tli RNaseH Plus) (Takara) and a CFX-96 Real-Time system (Bio-Rad). Primers were listed in Supplementary Table S5.

### In vitro assay of TET1 activity

A pMXs-based retrovirus encoding a short version of TET1 containing the catalytic domain (1397–2039 aa) was introduced into MEFs cultured in high glucose DMEM supplemented with 10% FBS, NEAA, and GlutaMAX. Three days after the infection, nuclear extraction was prepared with a Nuclear Extraction kit (Abcam). In total, 20 μg purified TET1 protein were used to replace the nuclear extraction when indicated.

Two oligonucleotides (forward 5′-CTCCTCAACTTCGATCACCGTCTC-3′ and Reverse 5′-GAGACGGTGATCGAAGTTGAGGAG-3′) complemented to each other were purchased from IGEbio and purified by HPLC. Biotin was labeled on the 5′ end of the reverse oligonucleotide. By controlling the methylation status of the CpG in the middle of the two oligonucleotides, full-methylated, hemi-methylated, and un-methylated CpG sites were prepared. Then, 40 pmol of annealed oligonucleotide was incubated with 50 μl of nuclear extract (approximately 50 μg total protein) in the presence of 1 mM α-ketoglutaric acid (Sigma), 100 μM Fe(NH_4_)_2_(SO_4_)_2_ (Sigma), 1 μM ATP (Sigma) and HEPES buffer (pH 8.0). The reactions were carried out at 37 ℃ for 24 h.

TET1 was deactivated by a 50 min incubation at 65 ℃. Then, 30 μl of Dynabeads^TM^ MyOne^TM^ Streptavidin C1 beads (Thermo Fisher) were used to purify the biotin-labeled and annealed oligonucleotides from the reaction system mentioned above. After washing the beads twice with 5 mM Tris-HCl (PH 7.5) supplemented with 0.5 mM EDTA and 1 mM NaCl, oligonucleotides were dissociated in 10 mM EDTA (PH 8.2) with 95% formamide. The dissociated oligonucleotides were purified with a QIAquick Nucleotide Removal kit (QIAGEN, cat. nos. 28304), and used for sequential digestion with nuclease P1 (Sigma, 45 ℃ for 4 h), phosphodiesterase I (Sigma, 37 ℃ for 2.5 h) and alkaline phosphatase (NEB, 37 ℃ for 3 h). The products were used for HPLC.

### Assays of DNA methylation

DNA was extracted using a Wizard Genomic DNA Purification Kit (Promega) according to the manufacturer’s instructions. The DNA methylation levels were determined using various methods.

*HPLC*. Purified DNA was digested with nuclease P1, phosphodiesterase I, and alkaline phosphatase. The nuclease P1 digestion reaction contained 5 μg of DNA, 5 μl of 100 mM NH_4_OAc, and 1 μl of Nuclease P1. The reaction mixture was kept in a 45 ℃ water bath for 12 h. Then, 6 μl of 1 M NH_4_HCO_3_ and 1 μl of phosphodiesterase I were added for a 12-h incubation at 37 ℃. 5 μl of Cutsmart Buffer and 1 μl of alkaline phosphatase were used for an additional 12-h incubation at 37 ℃. The resulting DNA digestion solution was analyzed by electrophoresis on a 1% agarose gel to verify that the digestion was complete.

The DNA digestion solution was diluted 3-fold and filtered through a 0.2-µm nylon membrane (Agilent). Each sample (10 μl) was loaded and analyzed on an Agilent 1260 BIO machine with a C18 reverse-phase column (2.1 × 50 mm, 1.8 μm, Agilent ZORBAX Eclipse Plus C18). The mobile phase consisted of 7 mM ammonium acetate pH 6.7/5% methanol (v/v); the flow rate was 0.3 ml per min, and the detector was set at 280 nm.

Calibration curves were generated using 2′-deoxycytidine (Sigma, D3897) and 5-methyl 2′-deoxycytidine (Chemcruz, sc278256). The concentrations of dC and 5mdC in the samples were calculated by interpolation from the calibration curves. The DNA methylation level was calculated as 5mdC/(dC + 5mdC) × 100%.

*WGBS*. MEFs were digested and fixed with 70% ethanol overnight. The fixed cells were treated with 0.25 mg/ml RNase A at 37 °C for 30 min to remove RNA and stained with 50 μg/ml propidium iodide (Sigma) for 30 min. The resulting cell suspension was used to enrich the cells in the G1/S-phases and S/G2-phases by FACS using a BD FACSAria II flow cytometer. Approximately 3 × 10^6^ cells in each phase of the cell cycle were sorted by FACS, and DNA was extracted from the sorted cells as described above. The purity of the DNA was determined using a K5500 spectrophotometer, and DNA quantification was performed using a Qubit® 3.0 fluorometer.

The purified DNA was shipped on dry ice to Annoroad Gene Technology Co. Inc., Beijing, China for WGBS. Bisulfite-seq DNA libraries were prepared using standard Illumina protocols. Briefly, the genomic DNA was fragmented by sonication to 100–300 bp, followed by blunting, 3′-end addition of dA, and adapter ligation according to the manufacturer’s instruction (Illumina). After the 3′A addition and adapter ligation, the DNA fragments were subjected to sodium bisulfite conversion using the ZYMO EZ DNA Methylation-Gold kit (ZYMO REASEARCH, USA). The bisulfite-treated DNAs were PCR amplified. The resultant DNAs were sequenced on an Illumina HiSeq2500 sequencer as paired-end 125-bp reads.

The raw data were trimmed with Trimmomatic and a quality analysis was performed with FastQC. Mapping was performed using Bismark to GRCm38 builds for the mouse genome^25^. All alignments were performed with high stringency allowing for only one base mismatch (*n* = 1), and the mapped data were deduplicated before the analyses. We only included cytosines that are covered by at least five reads. The resulting methylation profiles from germ cells covered up to 81% of the all cytosines genome-wide.

*RRBS*. MEFs were infected with retrovirus carrying the indicated genes twice as follows: the first infection on Day 0 and the second infection on Day 1. The cells used in the MEF experimental system (MEF) were cultured as normal MEFs for an additional 5 days. The cells used in the Vc-/Vc + OKMS reprogramming experimental systems (Vc-/Vc + ) were also infected with retrovirus carrying the four Yamanaka factors during the first two days and subjected to further reprogramming over the following 5 days.

Five days after infection, approximately 3 × 10^6^ cells per group were harvested by digestion with 0.25% trypsin. DNA was extracted from the cells, purified, and quantified as described above. The purified DNA was shipped on dry ice to Annoroad Gene Technology Co. Inc., Beijing, China for RRBS. RRBS was performed according to previously reported protocols^26^. Briefly, the genomic DNA was digested by restriction enzyme MspI. End repair was performed, and A and adapters in which the cytosines in the paired-end adapter sequence were methylated were added. The ligated product was subjected to size selection on a 2% agarose gel. Agarose gel bands with the inserted genomic DNA size 40–110 bp and inserted genomic DNA size 110–220 bp were excised, and two libraries were generated from each sample (one library consisting of 40–110 bp target sequences and the other library consisting of 110–220 bp target sequences). The DNA from the excised gel pieces was recovered with a QIAGEN Gel Extraction Purification Kit, followed by bisulfite treatment using a ZYMO EZ DNA Methylation-Gold kit. The resulting converted DNA was amplified by PCR and purified. The RRBS libraries were subjected to paired-end 50 nt sequencing with HiSeq 2500.

The raw sequencing data, clean reads, and methylation information of each cytosine that was sequenced at least once during RRBS were provided to the authors for further analysis.

### Calculation of AMDs

To obtain the absolute methylation difference (AMD) of individual CpG sites, the absolute value of the methylation difference between the positive and negative strands was calculated (Supplementary Fig. S2b). To obtain the AMD of individual gene, the methylation levels of CpG sites near TSS were first averaged on both the positive and negative stands before calculating the absolute value of the methylation difference between the two strands (Supplementary Fig. S2c). The AMDs of the CpG sites or genes in the G_1_ and G_2_/M phase were averaged to present the AMDs in the MEFs.

The theoretical AMD was calculated differently. First, we assumed that there were no hemi-methylated CpG sites. We divided all CpG sites detected in the current WGBS into 102 groups (0%, 0–1%, …, 99–100%, and 100%) based on their methylation levels, and their average methylation levels were close to 0%, 0.5%, …, 99.5%, and 100%, respectively. The sequencing depth of all CpG sites were summarized for each group. Then, we calculated the mathematical expectation of the absolute difference between two measurements of CpG sites with particular methylation levels and sequencing depth. For example, 5% of the CpG sites with methylation levels of 19–20% were sequenced at a depth of 10. The mathematical expectation of the absolute difference was 14.82%. Then, we summed these mathematical expectations and used this value as the theoretical AMD, since the positive and negative strands can be considered two separated measurements of one CpG site without considering hemi-methylation. The actual AMDs were significantly higher than the theoretical AMDs, highlighting the existence of hemi-methylated CpG sites.

The theoretical AMDs of particular genes were calculated by considering the CpG sites near the corresponding TSS one CpG site. The methylation level was the average of all CpG sites near TSS. The sequencing depth was the sum of the sequencing depth of all CpG sites. The theoretical AMDs were shown in Supplementary Fig. S2c is the average of all protein-coding genes.

### RNA-seq

RNA was extracted from the cells using TRIzol reagent (Thermo Fisher). Illumina mRNA-seq libraries were prepared for each RNA sample using a TruSeq RNA Sample Preparation Kit v2; the mRNA-seq libraries were then sequenced on an Illumina NextSeq 500 instrument with a NextSeq 500 Mid Output Kit v2. RNA-seq was performed as previously described^27^. Briefly, the reads were aligned to a transcriptome index generated from Ensembl annotations (v79), using RSEM (RNA-seq by Expectation-Maximization) to estimate the transcript abundances^28^. The RNA-seq data are expressed in units of GC-normalized tag counts^29^.

### ATAC-seq and ATAC-qPCR

ATAC-seq was performed as previously described^30,31^. 50,000 cells were trypsinized with 0.25% trypsin, washed with 50 ml of cold PBS and resuspended in 50 ml lysis buffer (10 mM Tris-HCl pH 7.4, 10 mM NaCl, 3 mM MgCl_2_, and 0.2% IGEPAL CA-630). The suspension of the nuclei was centrifuged for 10 min at 500×*g* at 4 ℃, then 50 ml of transposition reaction mix (25 ml TD buffer, 2.5 ml Tn5 transposase and 22.5 ml nuclease-free H2O) from a Nextera DNA Library Preparation Kit (FC-121-1031, Illumina) was added. The samples were incubated at 37 ℃ for 30 min and isolated using a MinElute Kit (QIAGEN). The ATAC-seq libraries were PCR amplified for the appropriate number of cycles and purified with a Qiaquick PCR (QIAGEN) column. The library concentration was measured with a KAPA Library Quantification kit. Finally, the ATAC library was sequenced on a NextSeq 500 using a NextSeq 500 High Output Kit v2 (150 cycles) (FC-404-2002, Illumina) according to the manufacturer’s instructions.

The raw data were trimmed with Trimmomatic and mapped mouse genome (mm10) with Bowtie2. Mapped data removed duplicate with samtools (rmdup). We removed mitochondrial sequences using ‘grep –v ‘chrM’. Biological replicates were merged, and peaks were called using dfilter^32^ (with the settings: −bs = 100 –ks = 60 –refine).

In order to avoid producing a large number of false negative peaks we use the re-calling peaks strategy as previously described^33^. In brief, we first collected the supersets of all the peaks and merged them with the centers less than 350 bp. Similarly, we incorporate all ATAC-seq unique reads into a superset sequence library, and then delete all “open” reads that overlap 1 bp in any of the superset’s ATAC-seq peaks. We then randomly selected 50 million reads as pseudo-inputs to represent a random background. The appropriate background for the peak from non-peak invocation is 0.2734, resulting in a false positive rate of 0.1% based on peak invocation in pseudo-input. All downstream analyses are based on this threshold of 0.2734, and if ATAC-seq is lower than that, it is labeled “closed” and above “open.”

Primers were designed near the TSS of selected genes as indicated in Fig. 4i. qPCR was performed with these primers in corresponding ATAC library. The qPCR results in the MEFs with *Tet1* over-expression were normalized against those in the MEFs with *sh-Dnmt1* over-expression. Primers were listed in Supplementary Table S5.

### Statistical analysis

All experiments were repeated at least five times (*n* ≥ 5), except for sequencing. The data were analyzed and compared using a two-tailed *t*-test, one-way ANOVA with Dunnett’s post-hoc test, or two-way ANOVA with Bonferroni’s post-hoc test. The error bars and “*n*” represent the standard deviation (standard error as indicated) and the number of independent experiments, respectively. “*”, “**”, and “***” represent significant differences (*P* < 0.05, *P* < 0.01, and *P* < 0.001, respectively) from the indicated control groups. The statistical information was listed in Supplementary Table S6. When groups of CpG sites in WGBS or RRBS were analyzed, the standard error was too small to be plotted.

### Data deposition

The WGBS, RRBS, and RNA-seq data were deposited in the Gene Expression Omnibus under accession numbers GSE92903, GSE93058, and GSE93416, respectively. The high-throughput sequencing data are available under SuperSeries accession number GSE93417 (https://www.ncbi.nlm.nih.gov/geo/query/acc.cgi?token = yvsdooakjfslrml&acc = GSE93417).

GSE10871 and GSE140121 were used to provide the gene expression profiles of the ESCs and MEFs^34–37^). GSE93029 and the ATAC-seq results of the MEFs were used to provide chromatin accessibility information.

## Electronic supplementary material

Supplementary Information

Supplementary Table S1

Supplementary Table S2

Supplementary Table S3

Supplementary Table S4

Supplementary Table S5

Supplementary Table S6

## Electronic supplementary material

**Supplementary Information** accompanies the paper at (10.1038/s41421-018-0074-6).
