# Supplementary material for: Hemi-methylated CpG sites connect Dnmt1-knockdown-induced and Tet1-induced DNA demethylation during somatic cell reprogramming
Source: Cell Discov. 2019 Feb 12;5:11. doi: 10.1038/s41421-018-0074-6 (PMC6370818; doi:10.1038/s41421-018-0074-6)
Supplement: Supplementary file 1 — Supplementary Information [file 41421_2018_74_MOESM1_ESM.pdf]

Supplementary Information For

Hemi-methylated CpG sites connect *Dnmt1*-knockdown- and *Tet1*-induced DNA demethylation during somatic cell reprogramming

Songwei He[1,#], Fuhui Wang[1,2,3,4,#], Yixin Zhang[1,2.3,4,#], Jinlong Chen[1,2,3,4], Lining Liang[1,2,3,4], Yuan Li[1,3,5], Mengdan Zhang[1,2,3,4], Xiao Yang[1,3,4,5], Hongshen Pang[6], Yingying Li[1,3], Xiaofen Huang[1,3], Dajiang Qin[1,2,3,4], Duanqing Pei[1,2,3,4,5], Hao Sun[1,2,3,4,*], Hui Zheng[1,2,3,4,5,*]

[#] These authors contributed equally to the manuscript

[*] Correspondence should be addressed to Hui Zheng & Hao Sun, #190 Kaiyuan Ave. Science City, Guangzhou, China, 510530, Tel.: 86-20-32015334; Fax: 86-20-32015231; Email: zheng_hui@gibh.ac.cn & sun_hao@gibh.ac.cn

This file contains:

Supplementary Figs. S1-4

Legends of Supplementary Table S1-6

Supplementary Figure S1

Two kinds of DNA demethylation share targets (related to Fig. 1)

(a) The influences of *sh-Dnmt1* and *Tet1* on methylation levels of all CpG sites detected in current RRBS were summarized.

(b-d) CpG sites with larger demethylation than average were further selected. The overlapped targets of the two kinds of demethylation were summarized in (b). In addition, the correlation between demethylation induced by *sh-Dnmt*1 and *Tet1* were listed in (c). Constantly demethylated CpG sites (about 165 thousand CpG sites, d) were summarized by overlapping the results in (b).

(e) *Dnmt1* and *sh-Dnmt1* were over-expressed with *Tet1*. Methylation levels of all CpG sites in genome were summarized.

Figure S1

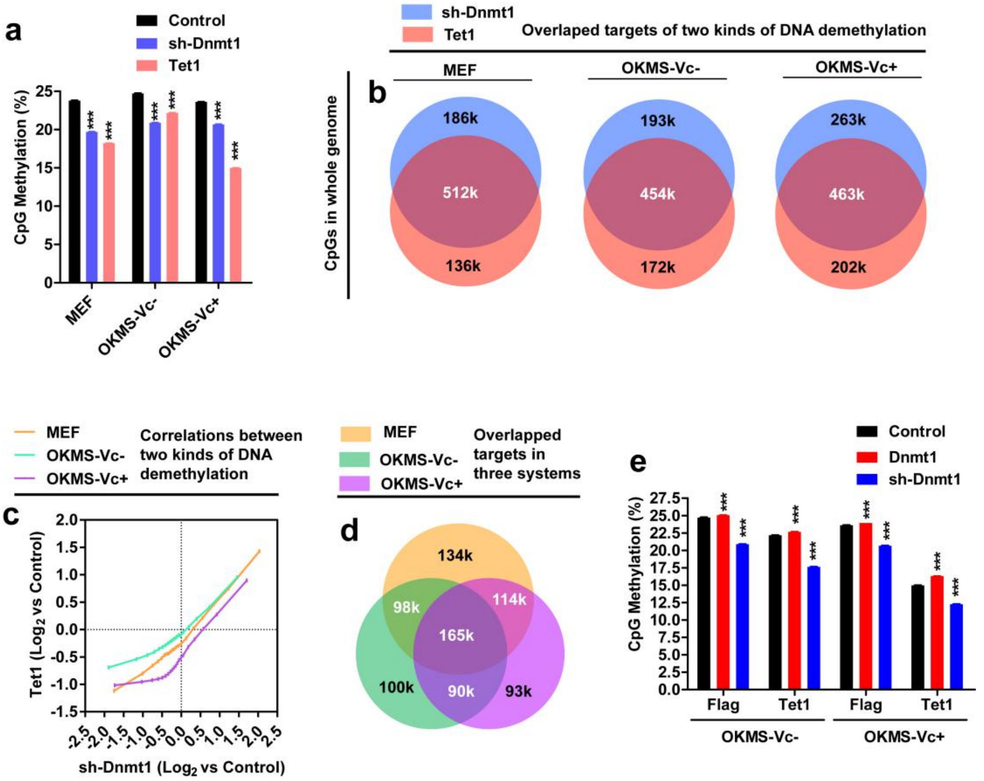

Supplementary Figure S2

DNMT1 counteracts with TET1 (related to Fig. 2)

(a) Schematic illustration of the generation of hemi-methylated CpG sites, and the four methylation reads of one particular CpG site obtained by performing WGBS.

(b-c) The methylation differences between indicated reads were averaged. Absolute methylation differences (AMDs) were calculated by averaging the absolute values of differences in methylation between positive and negative strands of all CpG sites (ave. of abs.[+vs-]). The theoretical expectation was calculated as described in Materials and Methods. Significant higher AMDs (both in G_1 and G_2 phase, and both in CpG and gene levels) than theoretical expectation confirmed the existence of hemi-methylated CpG sites.

(d-e) CpG sites and genes were sorted according to the enrichment of hemi-methylation (AMDs between two strands) and grouped into 14 and 20 groups, respectively. The average methylation levels of different groups were plotted against their AMDs.

(f-k) 20% CpG sites (around TSS, f, h, j) and genes (g, i, k) with higher enrichment of hemi-methylation (AMDs) were analyzed separately from the remaining 80%. The abilities of *sh-Dnmt*1 and *Tet1* to induce demethylation were listed in (f-g). The abilities of *Tet1* to promote *sh-Dnmt1*-induced demethylation were summarized in (h-i). The counteraction between *Dnmt1* and *Tet1* was listed in (j-k).

Figure S2

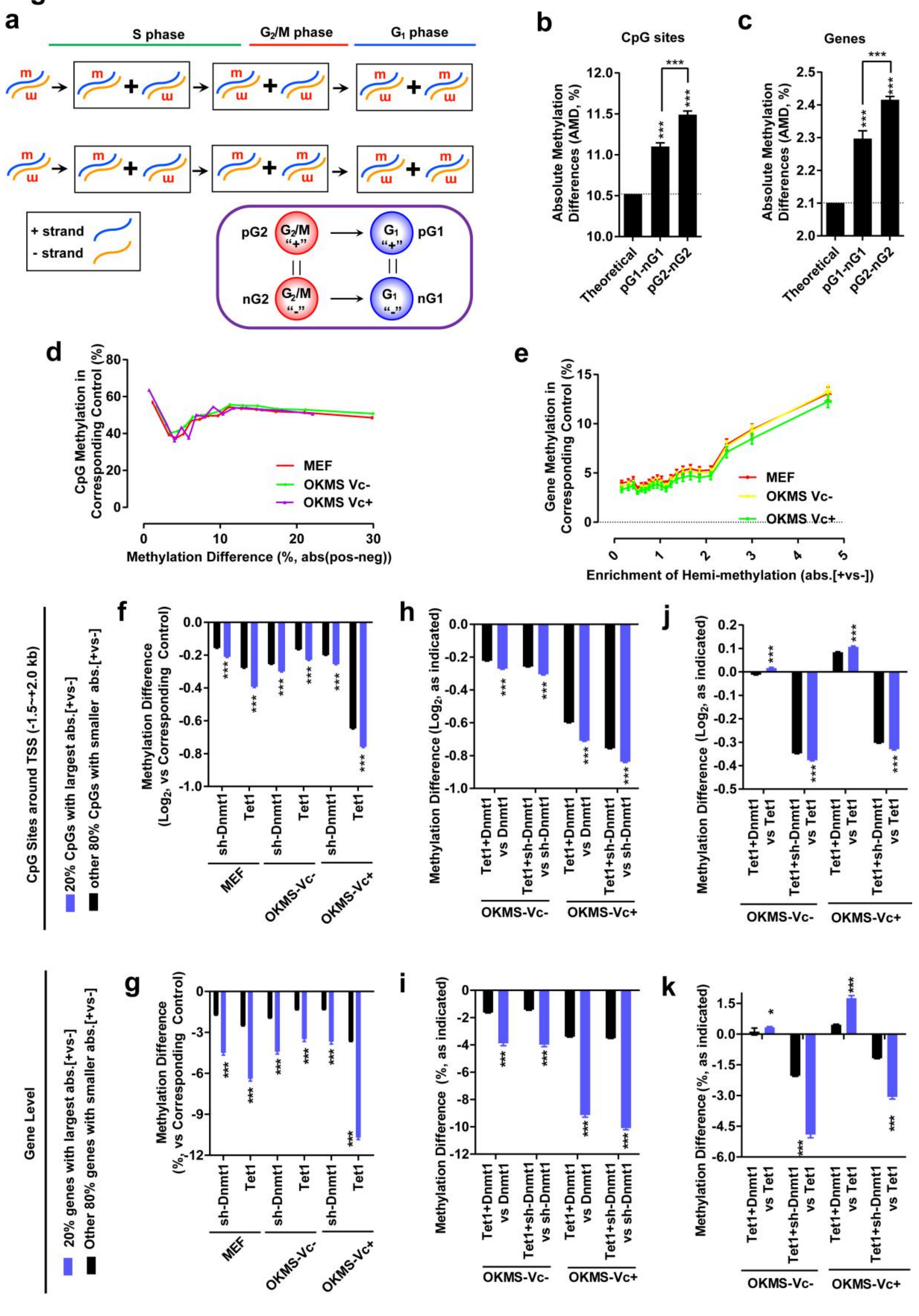

a
S phase
G2/M phase
G1 phase
+ strand
- strand
pG2
nG2
pG1
nG1
b
CpG sites
Absolute Methylation Differences (AMD, %)
Theoretical
pG1-nG1
pG2-nG2
c
Genes
d
CpG Methylation in Corresponding Control (%)
MEF
OKMS Vc-
OKMS Vc+
Methylation Difference (%, abs(pos-neg))
e
Gene Methylation in Corresponding Control (%)
Enrichment of Hemi-methylation (abs.[+vs-])
CpG Sites around TSS (-1.5~+2.0 kb)
20% CpGs with largest abs.[+vs-]
other 80% CpGs with smaller abs.[+vs-]
f
Methylation Difference (Log2, vs Corresponding Control)
sh-Dnmt1
Tet1
MEF
OKMS-Vc-
OKMS-Vc+
h
Methylation Difference (Log2, as indicated)
Tet1+Dnmt1 vs Dnmt1
Tet1+sh-Dnmt1 vs sh-Dnmt1
j
Tet1+Dnmt1 vs Tet1
Tet1+sh-Dnmt1 vs Tet1
Gene Level
20% genes with largest abs.[+vs-]
Other 80% genes with smaller abs.[+vs-]
g
Methylation Difference (%, vs Corresponding Control)
i
Methylation Difference (%, as indicated)
k

Supplementary Figure S3

Enrichments of hemi-methylation in specific genes (related to Fig. 5)

(a) CpG sites were divided into different groups basing on their methylation levels and distances from surrounding CpG sites. The enrichment of hemi-methylation (AMDs between two strands) was summarized. CpG sites with particular methylation levels in MEFs (20~85%) and distances from surrounding CpG sites (45~90 bp) were enriched with hemi-methylated CpG sites.

(b) Genes were divided into 10 groups based on their methylation levels. The enrichment of CpG sites with different levels of methylation was plotted for these ten groups of genes. CpG sites with particular methylation levels in MEFs (20~85%) were enriched in genes with methylation levels between 20% and 60%.

(c) Correlation between gene expression and methylation in MEFs were plotted. Particular levels of demethylation were used to simulate the influences of demethylation on gene expression. With similar demethylation, the closer to 60% of the methylation levels of genes the larger up-regulation was proposed.

Figure S3

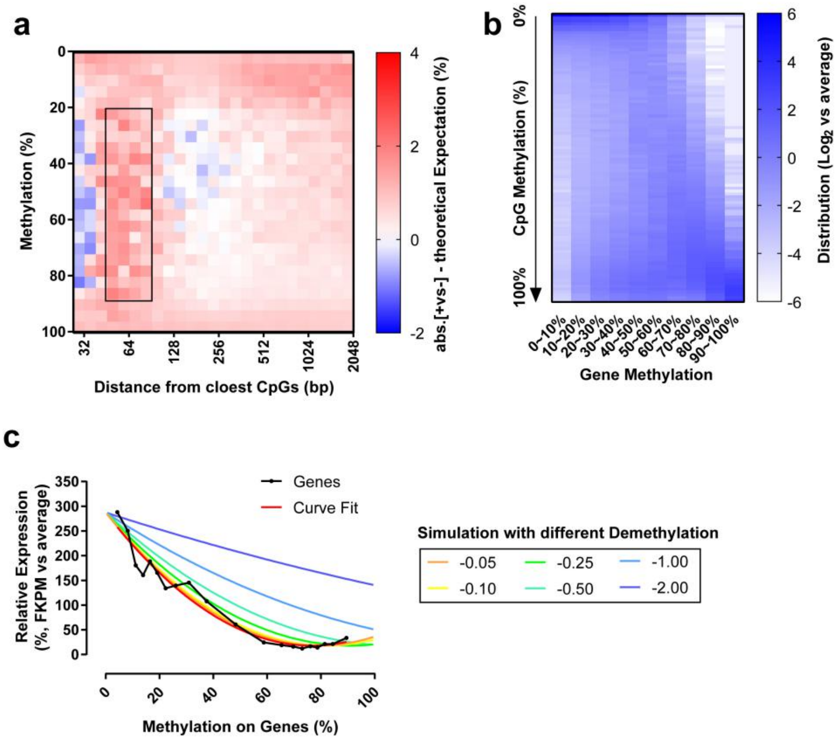
a
Methylation (%)
Distance from cloest CpGs (bp)
abs.[+vs-] - theoretical Expectation (%)
b
CpG Methylation (%)
0%
100%
Gene Methylation
0~10%
10~20%
20~30%
30~40%
40~50%
50~60%
60~70%
70~80%
80~90%
90~100%
Distribution (Log2 vs average)
c
Relative Expression (%, FKPM vs average)
Methylation on Genes (%)
Genes
Curve Fit
Simulation with different Demethylation
-0.05
-0.10
-0.25
-0.50
-1.00
-2.00

#

Supplementary Figure S4

Vc increases the ability of Tet1 to induce DNA demethylation (related to Fig. 6)

(a) HPLC was used to determine overall DNA methylation levels 3 days after over-expressing *Tet1* or *sh-Dnmt1*.

(b) The abilities of purified TET1 protein to demethylate hemi- and full-methylated CpG sites in the presence or in the absence of Vc.

(c-e) To confirm this, the demethylation on hemi-methylated CpG sites was determined by analyzing CpG sites with methylation levels between 0~10% and large AMDs between the two strands (top 10%). The demethylation on full-methylated CpG sites was determined by analyzing CpG sites with methylation levels between 60~90% and small AMDs between the two strands (top 10%). To incorporate the different expression of *Tet1* and *Dnmt1* in different experimental systems (c-d), the demethylation activities of *Tet1* or *sh-Dnmt1* were assumed at the same levels in MEFs and during reprogramming without Vc. As summarized in (e), the abilities of *sh-Dnmt1* to demethylate full- and hemi- methylated CpG sites were constant in all three experimental systems. The abilities of *Tet1* to demethylate hemi-methylated CpG sites increased by about 20% during reprogramming with Vc when compared with the other two systems. The abilities of *Tet1* to demethylate hemi-methylated CpG sites increased by about 100% in the presence of Vc.

Figure S4

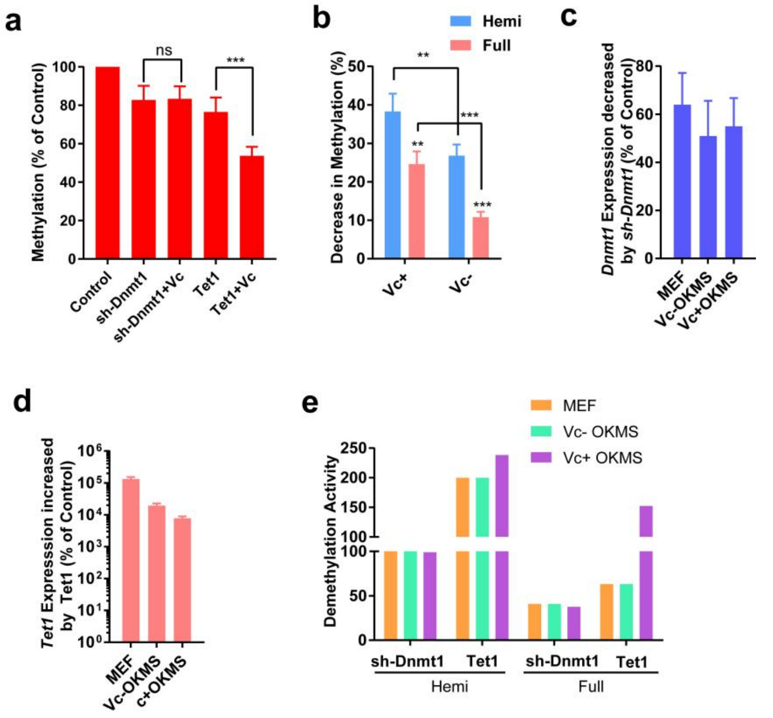
a
Methylation (% of Control)
ns
***
Control
sh-Dnmt1
sh-Dnmt1+Vc
Tet1
Tet1+Vc
b
Hemi
Full
Decrease in Methylation (%)
Vc+
Vc-
c
Dnmt1 Expresssion decreased by sh-Dnmt1 (% of Control)
MEF
Vc-OKMS
Vc+OKMS
d
Tet1 Expression increased by Tet1 (% of Control)
MEF
Vc-OKMS
c+OKMS
e
MEF
Vc- OKMS
Vc+ OKMS
Demethylation Activity
sh-Dnmt1
Tet1
Hemi
Full

Legends of Supplementary Tables

Supplementary Table S1

1680 genes that were constantly demethylated by both *sh-Dnmt1* and *Tet1* (related to Fig. 1)

As title.

Supplementary Table S2

Gene methylation and chromatin accessibility (related to Fig. 4)

Gene methylation was calculated by averaging the methylation levels of detected CpG sites around TSS (-1.5~+2.0kb). Chromatin accessibility was calculated by determining the average reading of ATAC-seq around TSS (-1.5~+0.2kb).

Supplementary Table S3

698 Genes with particular methylation levels and CpG densities (related to Fig. 5)

As title.

Supplementary Table S4

606 Genes whose expression were reversed by *Tet1* and Vc (related to Fig. 6)

As title.

Supplementary Table S5

Primer information (related to all Figures)

As title.

Supplementary Table S6

Statistic information (related to all Figures)

As title.

#
